# History and national survey on reflex hammers: which is *the chosen one* of Brazilian neurologists?

**DOI:** 10.1055/s-0043-1768697

**Published:** 2023-05-09

**Authors:** Caio César Diniz Disserol, Alex Tiburtino Meira, Carla Caroline Schramm, Gustavo Koiti Kondo, Gustavo Leite Franklin, José Luiz Pedroso, Orlando Graziani Povoas Barsottini, Hélio Afonso Ghizoni Teive

**Affiliations:** 1 Universidade Federal do Paraná, Hospital de Clínicas, Departamento de Medicina Interna, Serviço de Neurologia, Curitiba PR, Brazil.; 2 Universidade Federal da Paraíba, Departamento de Medicina Interna, João Pessoa PB, Brazil.; 3 Universidade Federal de São Paulo, Departamento de Neurologia, Divisão de Neurologia Geral e Ataxias, São Paulo SP, Brazil.

**Keywords:** Neurological Examination, Percussion, Neurology, History, Reflex, Stretch, Exame Neurológico, Percussão, Neurologia, História, Reflexo de Estiramento.

## Abstract

**Background** Percussion is an important part of the neurological examination and reflex hammers are necessary to obtain it properly.

**Objective** We aimed to review the historical aspects of the main reflex hammers and to define the favorite one of Brazilian neurologists.

**Methods** We searched original and review articles about historical aspects of the reflex hammers in Scielo and Pubmed and conducted an online survey to investigate the favorite reflex hammer of Brazilian neurologists.

**Results** In the first part, we describe the major milestones in the creation of the reflex hammers. Following, we exhibit the results of the online survey: Babinski-Rabiner was the most voted.

**Conclusions** The origins of the reflex hammers goes back long before their creation, from a basic clinical examination method: percussion. Since the description of deep tendon reflexes and the creation of percussion hammers, much has been improved in this technique. Among all the hammers surveyed, the Babinski-Rabiner was the chosen one by a significant portion of Brazilian neurologists.

## Resumo

**Antecedentes** A percussão é uma parte importante do exame neurológico e os martelos de reflexo são necessários para obtê-la adequadamente.

**Objetivo** Nós visamos revisar os aspectos históricos dos principais martelos de reflexo neurológico e definir qual é o preferido dos neurologistas brasileiros.

**Métodos** Procuramos artigos originais e artigos de revisão sobre os aspectos históricos dos martelos de reflexo na Scielo e no Pubmed, e conduzimos um questionário online para investigar qual é o preferido dos neurologistas brasileiros.

**Resultados** Na primeira parte, descrevemos os principais marcos na criação dos martelos de reflexo. Na sequência, expomos os resultados do questionário online: Babinski-Rabiner foi o martelo mais votado.

**Conclusões** A origem dos martelos de reflexos vem muito antes de sua criação, a partir de um método de exame clínico básico: a percussão. Desde a descrição dos reflexos tendinosos profundos e da criação de martelos de percussão, muito se aperfeiçoou sobre essa técnica. Dentre todos os martelos pesquisados, o de Babinski-Rabiner foi o escolhido por uma parcela significativa dos neurologistas brasileiros.

## INTRODUCTION

In 1945, Professor Wartenberg, the world-famous neurologist, stated in his seminal book *The Examination of Reflexes* that “testing of reflexes and their proper evaluation undoubtedly constitute the most important part of the neurologic examination.” 1 Percussion with a reflex hammer is useful in the neurological examination 1 2 3 4 as it helps the examiner to define the nature of the neurological disease. The technique has been used in medicine since the 1760s and was first adopted in neurological examinations in the 19 ^th^ century. 2 5 Many different reflex hammers have been designed over the years. We present a review of the history of the main types and investigate the favorite reflex hammer of Brazilian neurologists.

## METHODS

The authors searched on Pubmed and Scielo original and review articles about historical aspects of reflexes and reflex hammers with the following search terms: “reflexes”, “reflex hammer”, “history of reflexes”, “examination of reflexes” and “neurological examination”. Furthermore, we conducted an online survey to investigate the favorite reflex hammer of Brazilian neurologists. The survey depicted the following aspects: sex, age, neurology timing (resident or years as a neurologist), institution of residency training, favorite personal reflex hammer, reasons for that choice, and if it reflects the preferred choice of your institution. The online survey was made on Google Forms, the link to answer the questionnaire was sent by email and WhatsApp and the results were organized on an Excel spreadsheet.

## RESULTS AND DISCUSSION – HISTORICAL REVIEW

### Percussion

The first description of the use of clinical percussion dates from 1761. The Austrian physician Leopold Auenbrugger (1722-1809) used the technique that year to examine the chest and abdomen of patients for the presence of fluids. His inspiration came from observing winemakers, who thumped wine casks to estimate the wine volume. 5 By 1826 the technique was widespread in clinical practice, and in 1828 the Scottish physician David Barry (1781-1836) developed the first percussion hammer, inspired by the Swiss veterinary practice of percussing the skull of cattle to investigate the presence of hydatid cysts. 6

### The history of reflex hammers

**Figure 1:**
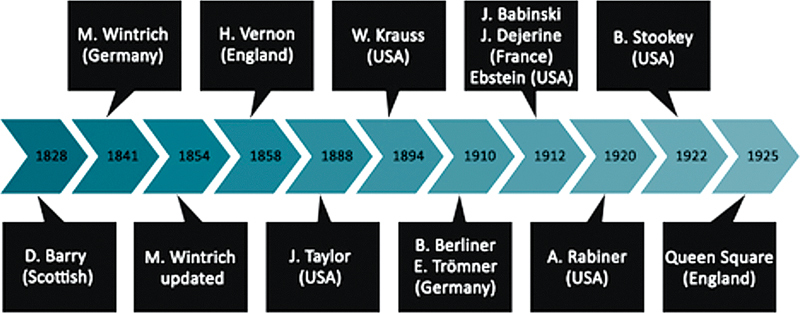
Timeline of the main reflex hammers.

**Figure 2:**
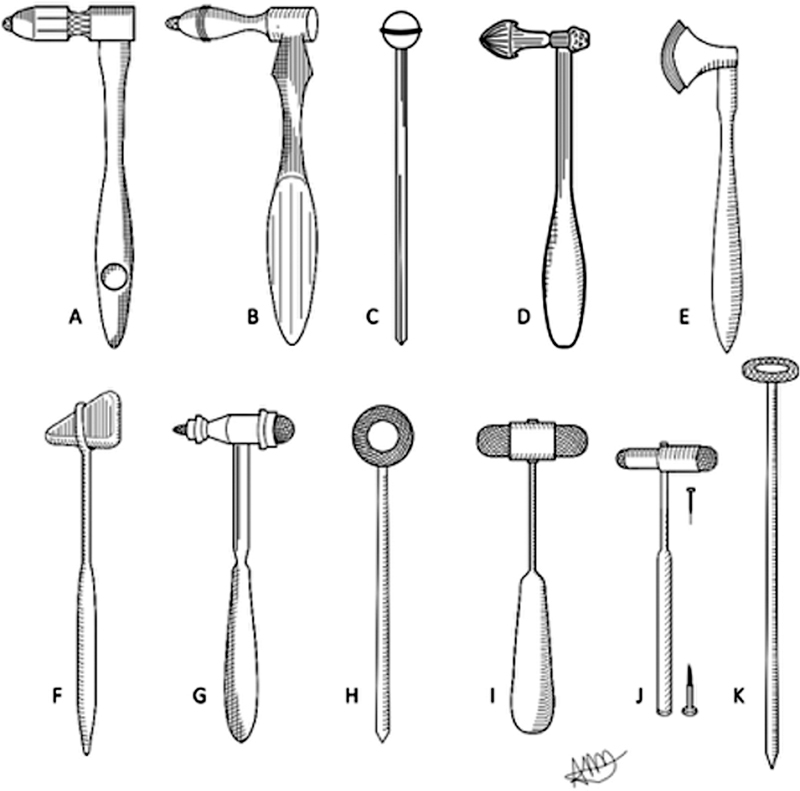
The first reflex hammers (A-E) and the more common hammers in use today (F-K). Illustrations are hand drawings made by one of the authors (ATM). Notes: A, Wintrich; B, Skoda; C, Vernon; D, Krauss; E, Berliner; F, Taylor; G, Trömner; H, Babinski-Rabiner; I, Dejerine; J, Buck; K, Queen Square.

In 1826, Pierre Adolphe Piorry (France, 1794-1879) described percussion with a device known as the pleximeter. Shortly after this, in 1828, the Scottish doctor David Barry developed a small hammer to strike the pleximeter. 5 In 1841, the German physician Max A. Wintrich (1812-1882) created what proved to be a popular hammer, and this was followed in 1854 by another hammer developed by Henry Vernon. 5 7 8 In 1875, in simultaneous publications, Wilhelm H. Erb and Carl F. O. Westphal described muscle stretch reflexes and the importance of the use of percussion hammers for assessing them. From then on percussion hammers were called reflex hammers and began to be produced on a commercial scale. 5 In the USA, two models were developed: the first by John Madison Taylor (1855-1931) in 1888, which was popularized by Silas Weir Mitchell, who introduced the graduation system to patellar reflex: KN + , KN + + and KN- 9 ; and the second by William C. Krauss (1863-1909), who incorporated gadgets to test temperature and touch sensations. 6 For many years Taylor's reflex hammer was featured in the American Academy of Neurology logo together with a tuning fork. 5 In 1910, Ernst L. O. Trömner (1868-1930) developed a reflex hammer with two percussion heads, a larger one for large tendons, and a smaller one for flexor tendons. In 1927, Henry W. Woltman (1889-1964) was greatly impressed by Trömner's hammer and bought several of them, both for his own use and for use by his colleagues at the Mayo Clinic, where he worked and where the hammer became a traditional professional item among neurologists. 5 In 1910, in Germany, Bernhard Berliner (1885-1976) developed a model with a heavier head and larger striking surface. 2 Also in Germany, in 1912, E. Ebstein modified Wintrich's hammer adding accessories to test sensation. 5 8 In 1912, in France, Joseph J. F. F. Babinski (1857-1932) developed two hammers. One had a disc-like head with a rubber ring around the edge, while in the other the disc was replaced by a rectangular plate, which also had a rubber ring around its edge. Shortly afterwards, in 1920, Abraham Rabiner (1892-1986) modified one of Babinski's hammers, making its head articulable allowing its use either parallel or perpendicular to the handle. This model became known as the Babinski-Rabiner hammer. 5 10 Concurrent, Joseph J. Dejerine (1849-1917) developed a reflex hammer with a cylindrical head made entirely of rubber. 6 In 1922, in the USA, the neurosurgeon Byron Stookey (1887-1966) developed his own hammer based on Wintrich's design. At that time, Stookey was examining patients with peripheral nerve injuries sustained during World War I. 11 The Queen Square hammer was developed around 1925 and was based on Vernon's hammer. Its name is a tribute to the National Hospital for Nervous Diseases, where it was invented by Miss Wintle, head nurse of the physiotherapy and radiology service. She wrapped a brass disk with a ring-shaped pessary and secured it to a bamboo rod to give a dense, flexible, lightweight, and painless hammer. 5 Other models, such as those designed by Buck and Henri Meige, were updated versions of the hammer developed by the Czech physician Josef Skoda (1805-1881), which Jean-Martin Charcot (1825-1893) considered the best hammer for obtaining the patellar reflex. 5 12 On the other hand, Gowers suggested eliciting the knee jerk by using the ulnar surface of the hand to strike the patellar tendon directly (Gower's maneuver). However, this was not considered acceptable by J. Babinski. 10 13 During his daily clinical activities, William Osler (1849- 1919) used the rim of his stethoscope to tap deep tendon reflexes. 14 The Queen Square hammer is the reflex hammer of choice of UK neurologists, while at the Mayo Clinic Trömner's hammer is the preferred choice. 5 Figure 1 depicts the evolution of creation of the main reflex hammers and Figure 2 their illustrations.

### Characteristics of reflex hammers

Taylor's hammer (1888; Figure 2-F ) was the first to be produced on a commercial scale. It was lightweight with a triangular-shaped rubber head and a short, flattened handle. The larger edge represented the ulnar surface of the hand while the smaller edge was used to obtain more subtle reflexes. Over time, it was noticed that the tip was better for assessing the plantar reflex. 5 The Krauss hammer (1894; Figure 2-D ) allowed assessing reflexes and sensations. The handle, which was made of rubber, became warm when rubbed, and could therefore be used to test thermal sensation, while at the larger extremity of the handle, there was a hidden removable brush, which could be used for testing tactile sensation. The head was made of metal and had a triangular-shaped tool for testing pain sensation. 5 Trömner's hammer (1910; Figure 2-G ) weighed about 100 grams and had an elongated head with different-sized rubbers at either head and a tapered, flat handle. Berliner's hammer (1910; Figure 2-E ) was a heavy tool made of nickel-plated metal. The head was hatchet-shaped and tapered at the tip and had an edge covered with rubber. The Babinski-Rabiner hammer (1920; Figure 2-H ) was made of nickel-plated steel and had a cylindrical handle 20 to 25 cm long. The head was circular, articulated, and covered with rubber. The Babinski-Rabiner hammer differed from the Queen Square hammer in the material used to make the handle and its length: the Babinski hammer was shorter and had a metallic handle, while the Queen Square hammer was longer and usually had a bamboo handle (nowadays it is plastic). 5

## RESULTS AND DISCUSSION – ONLINE QUESTIONNAIRE

According to *Conselho Federal de Medicina* there are 4,960 neurologists with a principal active and regular inscription in Brazil, 15 from which 3,813 are subscribed to *Academia Brasileira de Neurologia* (ABN). The sample constituted of 558 responders represents 11.2% of all Brazilian neurologists and 14.6% of those enrolled in the ABN.

The demographic characteristics of the participants are disposed in Table 1 .

**Table 1:** Demographic characteristics of the participants of the online survey

Characteristic		N	%
Sex	Male	316	57
	Female	238	43
Age (years)	< 30	84	15.1
	30–45	319	57.5
	45–60	80	14.4
	> 60	72	13
Neurology timing (years as neurologist)	Resident	54	9.7
	< 5	157	28.3
	5–10	121	21.8
	10–20	100	18.1
	> 20	122	22

**Figure 3:**
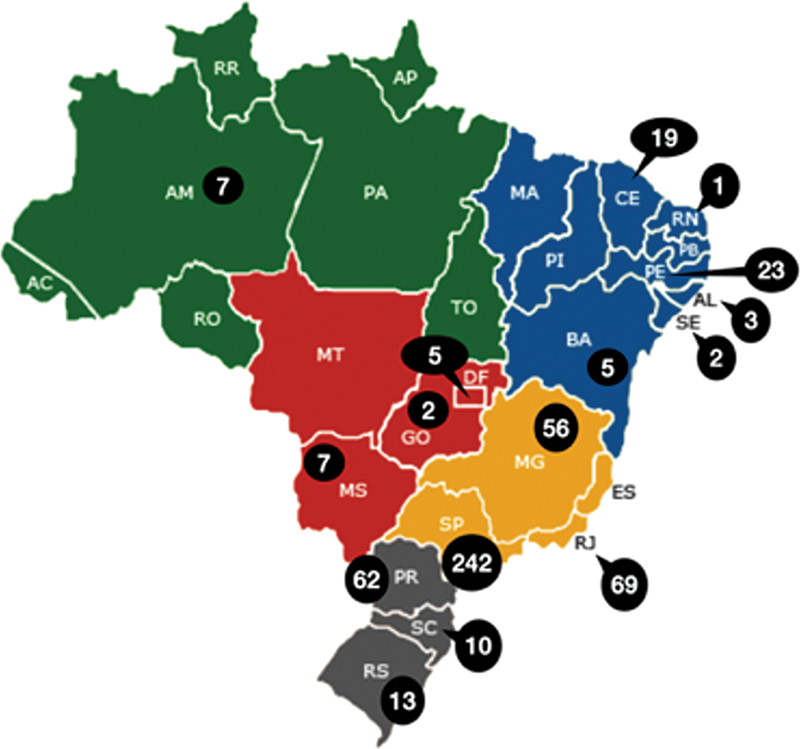
Brazilian region of residency training of the participants.

Figure 3 illustrates the Brazilian region of residency training of the responders. 530 participants answered this question.

**Figure 4:**
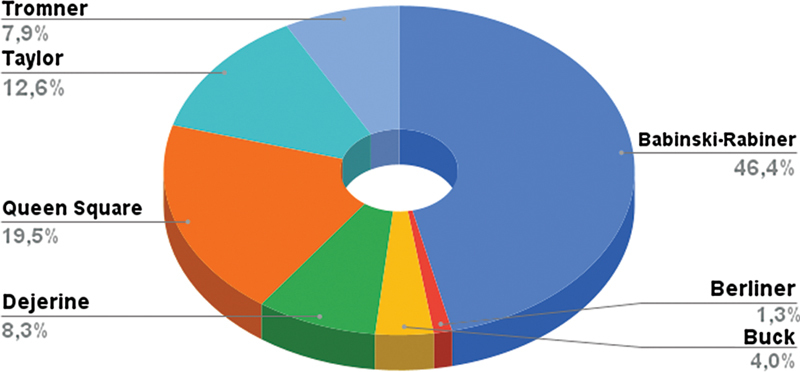
Favorite reflex hammers of Brazilian neurologists.

The frequency of reflex hammers chosen by the participants is disposed in Figure 4 . The Babinski-Rabiner was the favorite among Brazilian neurologists (257; 46%), and the Queen-Square was the second one (108; 19.3%). For those who chose the Babinski-Rabiner, 156 (60.7%) stated that it reflects their institution's choice.

Most of the responders (51.5%) consider that their choice did not reflect the preferred choice of their institution of residency training. For those that consider that their choice reflects the reflex hammer oriented by their chiefs in the institution where they were trained (48.6%), 42.4% stated that most neurologists of their institution use the same reflex hammer, and 6.2% stated that a minority use the preferred one. Buck was the most controversy: it is the favorite reflex hammer of 22 participants (3.9%), but only 3 stated that it is the preferred one of their institutions.

492 participants explained the reasons for their choice. Babinski-Rabiner's reflex hammer showed the following qualities: better individual adaptation and ergonomics, practical use, increased patient comfort, ease of evoking reflexes, heavy head weight, good weight distribution between the handle and the head, articulated head that facilitates transport and accessibility.

**Figure 5:**
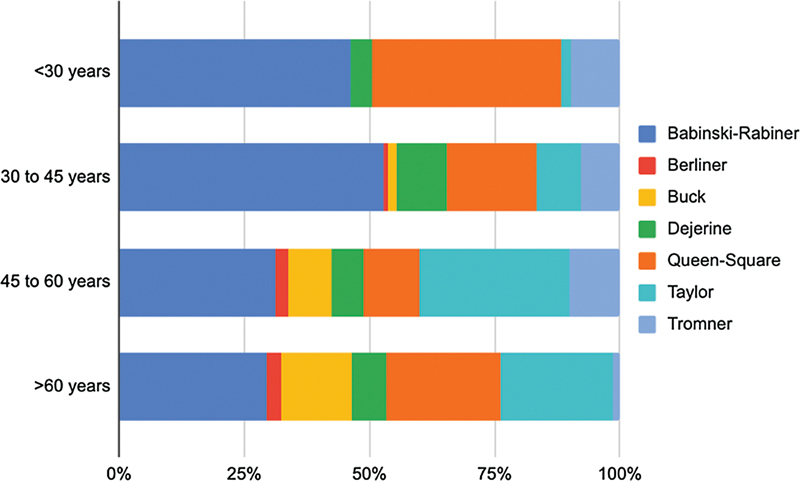
Reflex hammer's choice by age.

The reflex hammer's choice was homogeneous in all sets of ages ( Figure 5 ) and it was very similar between men and women.

In conclusion, percussion is a key point of the neurological examination. A wide range of reflex hammers is available nowadays, although many of the original models are no longer marketed. The choice of a hammer is left to the neurologist as no single model has been proven superior to the others.
